# Mortality of HIV-Infected Patients Starting Antiretroviral Therapy in Sub-Saharan Africa: Comparison with HIV-Unrelated Mortality

**DOI:** 10.1371/journal.pmed.1000066

**Published:** 2009-04-28

**Authors:** Martin W. G. Brinkhof, Andrew Boulle, Ralf Weigel, Eugène Messou, Colin Mathers, Catherine Orrell, François Dabis, Margaret Pascoe, Matthias Egger, for the International epidemiological Databases to Evaluate AIDS (IeDEA)

**Affiliations:** 1 Institute of Social and Preventive Medicine (ISPM), University of Bern, Switzerland; 2 Infectious Diseases Epidemiology Unit, School of Public Health and Family Medicine, University of Cape Town, South Africa; 3 Lighthouse Clinic, Lilongwe, Malawi; 4 Centre de Prise en Charge de Recherches et de Formation, Abidjan, Côte d'Ivoire; 5 Information, Evidence and Research Cluster, World Health Organization (WHO), Geneva, Switzerland; 6 The Desmond Tutu HIV Centre, Institute of Infectious Disease and Molecular Medicine, University of Cape Town, South Africa; 7 Institut de Santé Publique, d'Epidémiologie et de Développement (ISPED), Université Victor Segalen, Bordeaux, France; 8 Newlands Clinic, Harare, Zimbabwe; 9 Department of Social Medicine, University of Bristol, United Kingdom; San Francisco General Hospital, United States of America

## Abstract

**Background:**

Mortality in HIV-infected patients who have access to highly active antiretroviral therapy (ART) has declined in sub-Saharan Africa, but it is unclear how mortality compares to the non-HIV–infected population. We compared mortality rates observed in HIV-1–infected patients starting ART with non-HIV–related background mortality in four countries in sub-Saharan Africa.

**Methods and Findings:**

Patients enrolled in antiretroviral treatment programmes in Côte d'Ivoire, Malawi, South Africa, and Zimbabwe were included. We calculated excess mortality rates and standardised mortality ratios (SMRs) with 95% confidence intervals (CIs). Expected numbers of deaths were obtained using estimates of age-, sex-, and country-specific, HIV-unrelated, mortality rates from the Global Burden of Disease project. Among 13,249 eligible patients 1,177 deaths were recorded during 14,695 person-years of follow-up. The median age was 34 y, 8,831 (67%) patients were female, and 10,811 of 12,720 patients (85%) with information on clinical stage had advanced disease when starting ART. The excess mortality rate was 17.5 (95% CI 14.5–21.1) per 100 person-years SMR in patients who started ART with a CD4 cell count of less than 25 cells/µl and World Health Organization (WHO) stage III/IV, compared to 1.00 (0.55–1.81) per 100 person-years in patients who started with 200 cells/µl or above with WHO stage I/II. The corresponding SMRs were 47.1 (39.1–56.6) and 3.44 (1.91–6.17). Among patients who started ART with 200 cells/µl or above in WHO stage I/II and survived the first year of ART, the excess mortality rate was 0.27 (0.08–0.94) per 100 person-years and the SMR was 1.14 (0.47–2.77).

**Conclusions:**

Mortality of HIV-infected patients treated with combination ART in sub-Saharan Africa continues to be higher than in the general population, but for some patients excess mortality is moderate and reaches that of the general population in the second year of ART. Much of the excess mortality might be prevented by timely initiation of ART.

*Please see later in the article for Editors' Summary*

## Abstract 2

Comparing mortality rates between patients starting HIV treatment and the general population in four African countries, Matthias Egger and colleagues find the gap decreases over time, especially with early treatment.

## Editors' Summary

**Background:**

Acquired immunodeficiency syndrome (AIDS) has killed more than 25 million people since 1981 and more than 30 million people (22 million in sub-Saharan Africa alone) are now infected with the human immunodeficiency virus (HIV), which causes AIDS. HIV destroys immune system cells (including CD4 cells, a type of lymphocyte), leaving infected individuals susceptible to other infections. Early in the AIDS epidemic, most HIV-positive people died within ten years of infection. Then, in 1996, highly active antiretroviral therapy (ART)—combinations of powerful antiretroviral drugs—was developed and the life expectancy of HIV-infected people living in affluent countries improved dramatically. Now, in industrialized countries, all-cause mortality (death from any cause) among HIV-infected patients treated successfully with ART is similar to that of the general population and the mortality rate (the number of deaths in a population per year) among patients with HIV/AIDS is comparable to that among patients with diabetes and other chronic conditions.

**Why Was This Study Done?:**

Unfortunately, combination ART is costly, so although HIV/AIDS quickly became a chronic disease in industrialized countries, AIDS deaths continued unabated among the millions of HIV-infected people living in low- and middle-income countries. Then, in 2003, governments, international agencies and funding bodies began to implement plans to increase ART coverage in developing countries. By the end of 2007, nearly three million people living with HIV/AIDS in these countries were receiving ART—nearly a third of the people who urgently need ART. In sub-Saharan Africa more than 2 million people now receive ART and mortality in HIV-infected patients who have access to ART is declining. However, no-one knows how mortality among HIV-infected people starting ART compares with non-HIV related mortality in sub-Saharan Africa. This information is needed to ensure that appropriate health services (including access to ART) are provided in this region. In this study, the researchers compare mortality rates among HIV-infected patients starting ART with non-HIV related mortality in the general population of four sub-Saharan countries.

**What Did the Researchers Do and Find?:**

The researchers obtained estimates of the number of HIV-unrelated deaths and information about patients during their first two years on ART at five antiretroviral treatment programs in the Côte d'Ivoire, Malawi, South Africa, and Zimbabwe from the World Health Organization Global Burden of Disease (GBD) project and the International epidemiological Databases to Evaluate AIDS (IeDEA) initiative, respectively. They then calculated the excess mortality rates among the HIV-infected patients (the death rates in HIV-infected patients minus the national HIV-unrelated death rates) and the standardized mortality rate (SMR; the number of deaths among HIV-infected patients divided by the number of HIV-unrelated deaths in the general population). The excess mortality rate among HIV-infected people who started ART when they had a low CD4 cell count and clinically advanced disease was 17.5 per 100 person-years of follow-up. For HIV-infected people who started ART with a high CD4 cell count and early disease, the excess mortality rate was 1.0 per 100 person-years. The SMRs over two years of ART for these two groups of HIV-infected patients were 47.1 and 3.4, respectively. Finally, patients who started ART with a high CD4 cell count and early disease who survived the first year of ART had an excess mortality of only 0.27 per 100 person-years and an SMR over two years follow-up of only 1.14.

**What Do These Findings Mean?:**

These findings indicate that mortality among HIV-infected people during the first two years of ART is higher than in the general population in these four sub-Saharan countries. However, for patients who start ART when they have a high CD4 count and clinically early disease, the excess mortality is moderate and similar to that associated with diabetes. Because the researchers compared the death rates among HIV-infected patients with estimates of national death rates rather than with estimates of death rates for the areas where the ART programs were located, these findings may not be completely accurate. Nevertheless, these findings support further expansion of strategies that increase access to ART in sub-Saharan Africa and suggest the excess mortality among HIV-infected patients in this region might be largely prevented by starting ART before an individual's HIV infection has progressed to advanced stages.

**Additional Information:**

Please access these Web sites via the online version of this summary at http://dx.doi.org/10.1371/journal.pmed.1000066.

- Information is available from the US National Institute of Allergy and Infectious Diseases on HIV infection and AIDS
- HIV InSite has comprehensive information on all aspects of HIV/AIDS
- Information is available from Avert, an international AIDS charity on many aspects of HIV/AIDS including HIV and AIDS in Africa, providing AIDS drug treatment for millions, and on the stages of HIV infection
- The World Health Organization provides information about universal access to HIV treatment and about the Global Burden of Disease project (in several languages)
- More information about the International epidemiological Databases to evaluate AIDS initiative is available on the IeDEA Web site

## Introduction

The widespread use since 1996 of highly active antiretroviral therapy (ART) has substantially improved the prognosis of HIV-infected patients both in industrialised and low-income settings [1]. Recent studies from industrialised countries have suggested that all-cause mortality in patients successfully treated with ART might approach that of the general population, and that in many patients mortality rates are comparable to those associated with other chronic conditions, such as diabetes [2]–[6]. Such comparisons are important to gain a better understanding of the treated history of HIV infection, to monitor and predict the progress of the HIV/AIDS epidemic, and to plan health services in the era of potent ART.

As a result of scaling up of ART in low- and middle-income countries, 2.99 million people living with HIV/AIDS were estimated to be receiving treatment at the end of 2007, representing 31% of the estimated 9.6 million people in urgent need of treatment in these countries [7]. In sub-Saharan Africa, the number of patients on combination ART increased from 1.38 million to 2.12 million from 2006 to 2007. Although the immunological and virological responses to treatment in resource-limited countries can equal that in high-income settings [1],[8]–[10], mortality of patients starting ART has been substantially higher than in industrialised countries, particularly in the first few months of treatment [1],[10],[11]. To our knowledge, no studies have compared mortality among HIV-infected people starting ART in sub-Saharan Africa with the non-HIV–related background mortality.

We analysed data from a network of treatment programmes in sub-Saharan Africa to compare mortality rates observed in HIV-1–infected patients starting ART with non-HIV–related mortality in four countries in sub-Saharan Africa.

## Methods

### The International Epidemiological Databases to Evaluate AIDS

Analyses were based on cohorts participating in the West African and Southern African regions of the International epidemiological Databases to Evaluate AIDS (IeDEA) [12]. The databases are regularly updated; the November 2007 version was used for the present analysis. We restricted analyses to five large treatment programmes in four sub-Saharan African countries, including two treatment programmes in townships in the greater Cape Town metropolitan area, Khayelitsha [9] and Gugulethu [13], South Africa; the Lighthouse clinic in Lilongwe, Malawi [14]; the Centre de Prise en Charge de Recherches et de Formation (CEPREF)/Agence National de Recherches sur le Sida (ANRS) 1203 cohort from Abidjan, Côte d'Ivoire [15]; and the Connaught Clinic in Harare, Zimbabwe [16]. All patients aged 16 y or older who were ART-naïve at the start of ART were included. ART was defined as any combination of three antiretroviral drugs. Loss to follow-up was assumed in patients who were not known to have died and who were not seen for at least 1 y before closing the database for the present analysis. The local Ethics Committees of all clinics approved participation in IeDEA, which was also approved by the Cantonal Ethics Committee in Bern, Switzerland.

### Estimates of HIV-Free Background Mortality

Country-specific rates of all-cause mortality and HIV-free mortality by sex and 5-y age groups were obtained from the World Health Organization (WHO) Global Burden of Disease project [17]. Beginning with the year 1999, WHO has been producing annual life tables for all member states. A key use of these tables is the calculation of healthy life expectancy (HALE), the basic indicator of population health published each year in the World Health Report [18]. The methods used to estimate all-cause and cause-specific mortality have been described in detail elsewhere [19],[20]. Briefly, life tables based on vital registration data, corrected for under registration of deaths using demographic techniques, were used to estimate all-cause mortality in South Africa and Zimbabwe. In Côte d'Ivoire and Malawi data from other sources, such as census and surveys, were applied to a modified logit life-table model, using a global standard [20],[21]. For all four countries Joint United Nations Programme on HIVAIDS (UNAIDS) estimates of HIV/AIDS mortality were used, on the basis of epidemiological models and sentinel surveillance data on HIV seroprevalence [22].

### Multiple Imputation of Missing Individual Patient Data

Information on the CD4 count, clinical stage at the start of ART, and vital status at the last contact date was missing in some patients. Vital status was considered missing if the patient was not known to have died and the last date of information was less than 2 y after starting ART or before the administrative closure date of the cohort, whatever came first. We used multiple imputation by chained equation methods to impute missing information [23]. Multiple imputation included the outcome, i.e., whether or not a patient had died. Baseline CD4 cell count, clinical stage of disease, and survival time after censoring were imputed conditional on each other as well as on age and sex. All prediction equations included cohort, log age at start of ART, and sex. To optimise the imputation procedure we further included available clinical information on baseline viral load, total lymphocyte count, and haemoglobin; since females had lower haemoglobin levels the interaction between haemoglobin and sex was also fitted. Continuous variables were normalised prior to imputation modelling if needed, using log-transformation for age at start of ART and survival time, and square-root transformation for the baseline CD4. Interval censoring was used for baseline CD4 and survival time to ensure imputation values within the appropriate range. To impute survival time we used the complete follow-up history of all patients and used a log distribution to sample survival time after censoring in patients for which no death was recorded. The imputation of log survival time involved left-censoring at the date of last information, but no right-censoring. In the analysis we right-censored survival time at 2 y or at the closure date of the cohort. We created 20 imputed datasets in total. We analyzed imputed datasets using Poisson regression models (see below) to examine the association of time on treatment (months 1–3, 4–6, 7–12, and 13–24 after start of ART) and patient characteristics at baseline as risk factors of relative survival. Estimates of coefficients were derived by averaging, and appropriate standard errors were calculated using the within and between imputation standard errors of the estimates using Rubin's rules [23].

### Modelling of Standardised Mortality Ratios and Excess Mortality

We quantified mortality of HIV-infected patients on ART relative to the mortality in the general population using excess mortality and standardised mortality ratios (SMRs). The excess mortality risk is derived using an additive model, by subtracting age- and sex-specific HIV-unrelated mortality rates in the reference population from mortality in HIV-infected patients. SMRs are based on a multiplicative model and calculated as the ratio of the number of observed deaths over the expected deaths, using age- and sex-specific rates of HIV-unrelated mortality from the reference population. The SMR thus quantifies how much higher mortality is in HIV-infected patients compared to the reference population, but gives no indication of the excess mortality in absolute terms. Excess mortality and SMRs with 95% confidence intervals (CIs) were obtained from generalised linear models with a Poisson error structure, as described by Dickman and colleagues [24]. The expected number of deaths due to causes other than HIV d*_j_ for observation *j* was calculated by multiplying the person-time at risk *y*_j_ by the corresponding sex, age- (in 5-y age groups), and country-specific rates of HIV-free mortality. The excess mortality model assumes piecewise constant hazards λ_j_, implying a Poisson process for the number of deaths *d*_j_ in each interval. The generalised linear model with Poisson error structure for outcome *d*_j_ involves offset *ln (y*_j_ *)* and the user-defined link function *ln (μ*_ j_ − d*_j_ *)*, where *μ*_ j_ *= *λ_j_ *y*_j_. In SMR modelling *d*_j_ is modelled with offset ln (d*_j_ *)*. Robust standard errors were used to account for the clustering of data on treatment programme. Significance testing was by Wald tests.

Multivariable models were calculated for excess mortality on the 20 imputed datasets. The interpretation of the excess hazard ratios (eHRs) from these models is similar to that of the hazard ratio in the familiar Cox model. For example, an eHR of 0.80 for females relative to males would indicate that females have a 20% lower risk of death as compared to males, after controlling for the variation in background mortality. The following variables were included: age, sex, ART regimen, baseline CD4 cell count, clinical stage of disease, and calendar period of starting ART. Time periods considered were months 1–3, 4–6, 7–12, and 13–24 after start of ART. ART regimen was defined as non-nucleoside reverse transcriptase inhibitor (NNRTI)-based, protease inhibitor (PI)-based, and other. Baseline CD4 count was analysed in five categories (0–24, 25–49, 50–99, 100–199, and 200 or more cells/µl). Clinical stage of disease was defined as less advanced (WHO stage I or stage II) or advanced (WHO stage III or stage IV). In a sensitivity analysis we excluded two sites with high loss to follow-up. All analyses were done in Stata version 10.0 (Stata Corporation), using the “ice” routine for imputation of missing values.

## Results

### Treatment Programmes and Patient Characteristics

The combined dataset included 13,249 patients. Table 1 describes the five treatment programmes from four sub-Saharan African countries. Patient numbers ranged from 857 patients (Connaught clinic, Zimbabwe) to 4,710 patients (Lighthouse clinic, Malawi). The majority of patients in each of the treatment programmes were women, the median age ranged from 32 to 37 y. The median baseline CD4 cell count ranged from 87 cells/µl in Khayelitsha, South Africa to 131 cells/µl in Abidjan, Côte d'Ivoire, and the proportion with advanced clinical stage of disease (WHO stage III/IV) from 68% (Connaught) to 90% (Khayelitsha). A total of 1,177 deaths were recorded during 14,695 person-years of follow-up. Crude estimates of cumulative mortality at 2 y on ART ranged from 7.4% to 12.3%, and loss to follow-up from 7.1% to 31.7% across programmes.

**Table 1:** Description of treatment programmes included in analyses.

Programme	Country	Number of Patients	Median Age (IQR)	Number of Women (%)	Median (IQR) Baseline CD4 (Cells/µl)	Number in WHO stage III/IV at Baseline (%^a^)	Number of Patients Lost to Follow-up at 2 y	Cumulative Loss to Follow-up (95% CI) at 2 y (%)^b^	Cumulative Mortality (95% CI) at 2-y (%)	
									Crude	Following Multiple Imputation^c^
**CEPREF**	Côte d'Ivoire	2,400	35 (30–42)	1,770 (74)	131 (51–217)	1,939 (82)	218	13.7 (12.1–15.6)	10.6 (9.3–12.1)	11.2 (9.7–12.8)
**Connaught**	Zimbabwe	857	37 (32–44)	585 (68)	102 (51–159)	263 (68)	33	7.1 (5.1–9.8)	7.4 (5.7–9.6)	7.5 (5.6–9.6)
**Gugulethu**	South Africa	1,916	33 (29–39)	1,310 (68)	103 (50–160)	1,528 (80)	62	7.6 (5.9–9.7)	11.1 (9.5–13.1)	11.1 (9.2–12.8)
**Khayelitsha**	South Africa	3,366	32 (28–38)	2,353 (70)	87 (35–146)	3,018 (90)	148	7.1 (6.1–8.4)	11.2 (10.1–12.4)	11.3 (10.2–12.5)
**Lighthouse**	Malawi	4,710	36 (30–42)	2,813 (60)	126 (54–211)	4,063 (86)	829	31.7 (29.9–33.6)	12.3 (11.1–13.6)	13.2 (11.9–14.4)
**Combined**	—	13,249	34 (29–41)	8,831 (67)	107 (46–175)	10,811 (85)	1,290	16.2 (15.4–17.1)	11.1 (10.5–11.8)	11.7 (11.1–12.3)

Number of patients (%) unless otherwise indicated.

a Percent of patients with known clinical stage at baseline.

b Estimated for patients with at least one additional potential year of follow-up until administrative censoring date of the database of their programme.

c Outcomes imputed in patients lost to follow-up.

CEPREF, Centre de Prise en Charge de Recherches et de Formation/Agence National de Recherches sur le Sida (ANRS) 1203 cohort.

Information on the CD4 count and clinical stage at the start of ART was missing for 2,535 patients (19.1%) and 529 patients (4.0%), respectively. Total follow-up time after imputation increased to 17,480 y, and the number of deaths to 1,338. Mortality estimates at 2 y were somewhat higher after imputation for the Centre de Prise en Charge de Recherches et de Formation (CEPREF) and Lighthouse cohorts, but similar to the crude estimates in the other cohorts (Table 1). Patient characteristics at baseline and the effect of multiple imputation of missing information on the distribution of CD4 cell count and clinical stage of disease at baseline are shown in Table 2. At 6 mo, the median CD4 cell count had increased to 245 cells/µl (interquartile range [IQR] 167–347), varying between 220 and 272 cells/µl across programmes. At 12 mo, the median CD4 count was 285 cells/µl (IQR 197–393), ranging from 253 to 307 cells/µl.

**Table 2:** Baseline characteristics and mortality over the first 2 y of ART.

Category	Subcategory	Original Data			Following Multiple Imputation^a^		
		*n* (%)	Person-Years	*n* Deaths (%)	*n* (%)	Person-Years	*n* Deaths (%)
**Overall**	**—**	13,249 (100)	14,695	1,177 (100)	13,249 (100)	17,480	1,338 (100)
**Age (y)**	**16–29**	3,436 (26)	3,856	276 (23)	3,436 (26)	4,564	309 (23)
—	**30–39**	5,875 (44)	6,567	521 (44)	5,875 (44)	7,789	594 (44)
—	**40–49**	2,919 (22)	3,232	266 (23)	2,919 (22)	3,851	308 (23)
—	**≥50**	1,019 (8)	1,041	114 (10)	1,019 (8)	1,276	127 (10)
**Sex**	**Female**	8,831 (67)	10,047	701 (60)	8,831 (67)	11,796	789 (59)
—	**Male**	4,418 (33)	4,648	476 (40)	4,418 (33)	5,684	549 (41)
**Initial ART regimen**	**NNRTI-based**	11,325 (85)	12,616	1,027 (87)	11,325 (85)	14,969	1162 (87)
—	**PI-based**	94 (1)	124	8 (1)	94 (1)	148	9 (1)
—	**Unknown or other**	1,830 (14)	1,955	142 (12)	1,830 (14)	2,363	167 (12)
**Baseline CD4 count (cells/**µ**l)**	**<25**	1,670 (13)	1,753	322 (27)	1,937 (15)	2,414	415 (31)
—	**25–49**	1,188 (9)	1,303	162 (14)	1,457 (11)	1,884	225 (17)
—	**50–99**	2,174 (16)	2,571	182 (15)	2,725 (21)	3,737	275 (20)
—	**100–199**	3,812 (29)	4,408	208 (18)	4,645 (35)	6,309	305 (23)
—	**≥200**	1,870 (14)	1,845	78 (7)	2,485 (19)	3,136	118 (9)
—	**Missing** a	2,535 (19)	2,815	225 (19)	—	—	—
**Clinical stage of disease**	**Less advanced**	1,909 (14)	2,125	53 (5)	2,079 (16)	2,800	65 (5)
—	**Advanced**	10,811 (82)	11,852	1,097 (93)	11,170 (84)	14,680	1,273 (95)
—	**Missing** a	529 (4)	718	27 (2)	—	—	—

a Multiple imputation was used to impute missing values of baseline CD4 count and clinical stage, and to impute outcomes in patients lost to follow-up (see Table 1).

Abbreviations: NNRTI, non-nucleoside reverse transcriptase inhibitor; PI, protease inhibitor.

### All-Cause, HIV-Associated, and HIV-Free Mortality in the General Population

**Figure 1:**
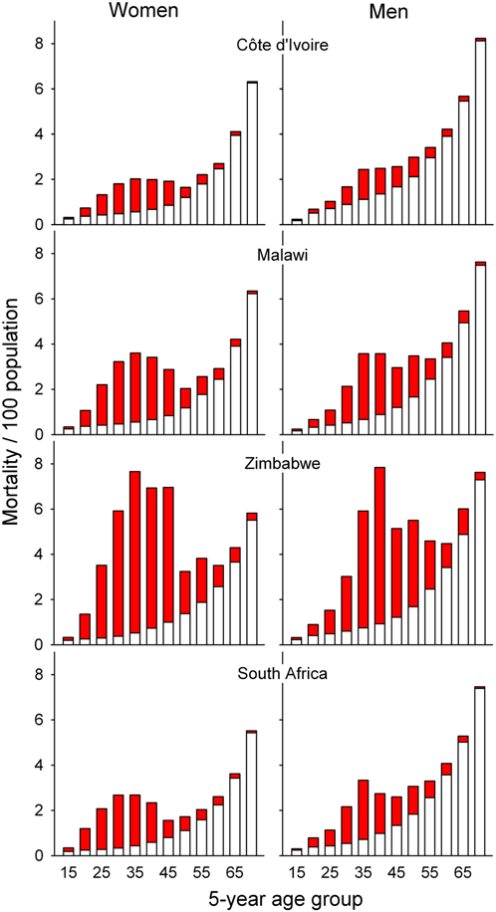
All-cause mortality for the year 2004 as the total of HIV-related (red area) and HIV-unrelated mortality (white area) by 5-y age group in Côte d'Ivoire, Malawi, South Africa, and Zimbabwe. Age groups are indicated by the lower age, e.g., age group 15 indicating age 15–19 y, age group 20 indicating age 20–24 y, and so forth. Data from the Global Burden of Disease Study [17],[20].

Figure 1 shows estimated all-cause mortality for the year 2004 as the sum of HIV-associated and HIV-free mortality by 5-y age group by country. Between age 20 and 50 y (the age range including 90% of patients starting ART in this study), the estimated relative contribution of HIV-associated mortality to all-cause mortality was 52% in Côte d'Ivoire, 74% in Malawi, 71% in South Africa, and 84% in Zimbabwe. Across all age groups, estimates of HIV-unrelated mortality were consistently higher in men than in women. The average mortality ratio for men versus woman aged between 20 and 50 y was 1.8 in Côte d'Ivoire, 1.2 in Malawi, 1.6 in South Africa, and 1.5 in Zimbabwe. The rates of HIV-unrelated mortality by 5-y age group and sex were used to model the mortality of HIV-infected patients on ART relative to the mortality in the general population. These rates are presented in Table S1.

### Risk Factors for Excess Mortality in HIV-Infected Patients Starting ART

The adjusted risk of excess mortality steeply declined with time period on ART. With reference to the first 3 mo, the eHR for the second year on treatment was 0.10, indicating a risk reduction of 90% (Table 3). Over the 2-y study period females were at 16% lower risk of excess mortality than males (eHR 0.84). There was strong evidence for a decline in excess risk with increasing baseline CD4 count: patients starting with a CD4 count of 200 cells/µl or more experienced an 81% reduction in risk over 2 y as compared to patients that started with a CD4 count of less than 25 cells/µl (eHR 0.19). Similarly, the excess risk was reduced by 72% (eHR 0.28) over the 2 y in patients starting with less advanced disease (WHO stage I/II) compared to patients starting with advanced disease (WHO stage III/IV). There was little evidence for an association between excess mortality and age, treatment regimen, or calendar period.

**Table 3:** eHRs for death according to different time periods after starting ART, demographic and clinical characteristics at baseline, and calendar period of starting ART.

Period, Characteristics, and Starting Period	eHR (95% CI)	*p*-Value
**Period**	—	<0.0001
Month 1–3	1	—
Months 4–6	0.36 (0.33–0.40)	—
Months 7–12	0.18 (0.15–0.22)	—
Months 13–24	0.10 (0.086–0.12)	—
**Age (y)**	—	0.34
16–29	1	—
30–39	1.06 (0.80–1.41)	—
40–49	1.09 (0.88–1.34)	—
≥50	1.27 (0.96–1.67)	—
**Sex**	—	0.044
Male	1	—
Female	0.84 (0.71–0.99)	—
**Initial regimen**	—	0.86
Two NRTIs + one NNRTI	1	—
Two NRTIs + one PI	1.04 (0.30–3.68)	—
Unknown or other combination	0.94 (0.73–1.21)	—
**Baseline CD4 (cells/**µ**l)**	—	<0.0001
<25	1	—
25–49	0.68 (0.58–0.81)	—
50–99	0.42 (0.32–0.56)	—
100–199	0.28 (0.22–0.34)	—
≥200	0.19 (0.14–0.26)	—
**Clinical stage**	—	<0.0001
WHO stage I/II	0.28 (0.17–0.46)	—
WHO stage III/IV	1	—
**Calendar period of starting ART**	—	0.24
Before 2005	1	—
2005 or later	1.16 (0.91–1.49)	—

eHRs from multivariable Poisson regression models, comparing mortality among HIV-infected patients, taking into account the background mortality among non-HIV–infected individuals in the general populations of the four countries included in the study. Models were adjusted for all variables listed in the table. *p*-Values are from Wald tests. eHRs for demographic and clinical characteristics at baseline, and calendar period of starting ART, are based on mortality over the first two years of treatment.

Abbreviations: NNRTI, non-nucleoside reverse transcriptase inhibitor; PI, protease inhibitor.

We examined effect modifications by adding interaction terms for variables time after starting ART, baseline CD4, and clinical stage of disease to the model. There was evidence that the effect of baseline CD4 count depended on the time period after starting ART (test of interaction, *p*<0.001), but not for the other possible interactions (*p*>0.48). The association between the baseline CD4 count and excess mortality became weaker with time on treatment and the interaction was included in estimating excess mortality and SMRs.

### Excess Mortality

**Figure 2:**
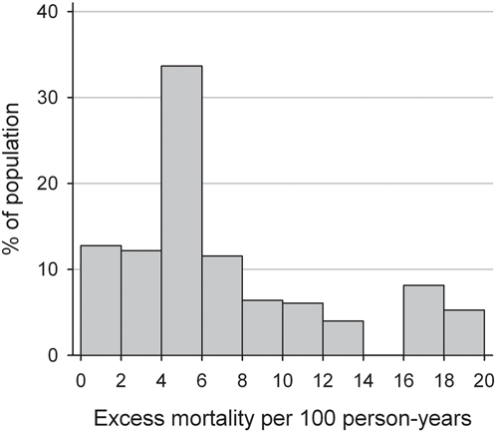
Distribution of excess mortality over the first 2 y of ART in patients starting ART in five treatment programmes in sub-Saharan Africa.

Excess mortality declined with time on treatment and increasing baseline CD4 cell count. It was lower in women as compared to men, and higher in patients starting ART with advanced stage of disease (Table 4). Overall excess mortality per 100 person-years was 6.95 (5.95–8.13), varying between 17.51 (14.50–21.14) and 1.00 (0.55–1.81) for patients starting with worst prognosis (CD4 cell count <25 cells/µl and advanced stage of disease) and best prognosis (CD4 cell count ≥200 cells/µl and less advanced stage of disease), respectively. In the second year on ART excess mortality in the patients group with best prognosis was 0.27 (0.08–0.94) per 100 person-years. Figure 2 shows the distribution of estimated excess mortality rates over the first 2 y of ART, taking into account baseline CD4 count, clinical stage, age, and sex. 34% of patients were exposed to excess mortality rates between four and six additional deaths per 100 person-years, 25% to rates below four per 100 person-years, and 41% to rates above six per 100 person-years. Table S2 gives excess mortality rates over 2 y by baseline CD4 count, clinical stage, and by sex and age group.

### Standardised Mortality

**Table 4:** Excess mortality per 100 person-years by time period on ART, baseline CD4 count, and clinical stage of disease.

CD4 Count (Cells/µl)	Clinical Stage	Time after Starting ART (mo)				
		1–3	4–6	7–12	13–24	Overall (1–24)
**<25**	**Advanced**	63.79 (44.67–91.10)	15.87 (8.62–29.22)	7.99 (4.87–13.09)	4.94 (3.39–7.21)	17.5 (14.5–21.1)
**—**	**Less advanced**	18.25 (9.15–36.41)	4.54 (1.91–10.79)	2.29 (0.96–5.43)	1.41 (0.75–2.67)	4.87 (2.64–9.00)
**25–49**	**Advanced**	38.32 (25.15–58.38)	14.36 (8.49–24.28)	7.87 (3.98–15.57)	2.70 (1.18–6.16)	12.1 (9.09–16.0)
**—**	**Less advanced**	10.96 (5.34–22.50)	4.11 (1.81–9.35)	2.25 (0.83–6.11)	0.77 (0.26–2.28)	3.36 (1.74–6.49)
**50–99**	**Advanced**	21.86 (11.39–41.97)	8.80 (4.46–17.36)	4.55 (2.22–9.33)	3.18 (1.65–6.12)	7.38 (4.98–10.95)
**—**	**Less advanced**	6.25 (2.49–15.70)	2.52 (0.98–6.44)	1.30 (0.45–3.77)	0.91 (0.36–2.29)	2.05 (0.98–4.31)
**100–199**	**Advanced**	13.73 (7.20–26.19)	7.52 (4.65–12.18)	2.71 (1.54–4.79)	1.81 (0.94–3.50)	4.83 (3.56–6.56)
**—**	**Less advanced**	3.93 (1.57–9.84)	2.15 (0.95–4.87)	0.78 (0.34–1.80)	0.52 (0.23–1.17)	1.35 (0.70–2.59)
**≥200**	**Advanced**	10.20 (7.55–13.77)	3.50 (1.90–6.45)	3.12 (0.19–5.01)	0.96 (0.37–2.49)	3.59 (2.82–4.56)
**—**	**Less advanced**	2.92 (1.53–5.58)	1.00 (0.43–2.35)	0.89 (0.48–1.66)	0.27 (0.08–0.94)	1.00 (0.55–1.81)
**Overall**	**Overall**	21.20 (19.21–23.38)	7.58 (6.48–8.86)	3.79 (2.93–4.90)	2.15 (1.79–2.58)	6.95 (5.95–8.13)

Results from Poisson model that included all variables listed and allowed for interaction between baseline CD4 cell count and time after starting ART.

SMRs, overall and stratified by time period on ART, baseline CD4 cell count, and clinical stage of disease are shown in Table 5. The overall SMR over the first 2 y was 18.7 (17.7–19.8), declining from 130.0 (110.9–152.4) to 4.0 (3.3–5.0) over months 1–3 to months 13–24, respectively. Over the first 3 mo, SMRs varied between 552.7 (400.1–763.5) for patients starting ART with worst prognosis to 30.2 (15.7–58.0) among patients starting with best prognosis. In the second year on ART, SMRs for these two patients groups were 11.5 (7.95–16.7) and 1.14 (0.47–2.77), respectively. Over the full first 2 y and depending on CD4 count and clinical stage of disease, SMRs varied between 47.1 (39.1–56.6) and 3.4 (1.9–6.2). Table S3 gives SMRs over 2 y by baseline CD4 count, clinical stage, and by sex and age group.

**Table 5:** SMRs by time period on ART, baseline CD4 count, and clinical stage of disease.

CD4 Count (Cells/µl)	Clinical Stage	Time Period (mo)				
		1–3	4–6	7–12	13–24	Overall (1–24)
**<25**	**Advanced**	552.7 (400.1–763.5)	142.7 (85.3–238.7)	37.2 (22.3–62.0)	11.5 (7.95–16.7)	47.1 (39.1–56.6)
	**Less advanced**	186.3 (99.3–349.2)	48.1 (22.7–102.0)	12.5 (5.52–28.4)	3.88 (2.10–7.17)	15.8 (8.99–27.9)
**25–49**	**Advanced**	333.1 (233.3–475.5)	130.4 (79.9–212.6)	37.2 (20.1–68.9)	7.01 (3.51–14.0)	31.4 (26.1–37.7)
	**Less advanced**	112.3 (59.9–210.4)	43.9 (20.7–93.1)	12.5 (5.17–30.3)	2.36 (0.95–5.85)	10.6 (6.08–18.4)
**50–99**	**Advanced**	192.2 (108.5–340.5)	80.4 (44.5–145.1)	22.6 (11.7–43.6)	8.04 (4.73–13.7)	19.6 (15.1–25.5)
	**Less advanced**	64.8 (28.9–145.0)	27.1 (11.9–61.7)	7.61 (2.95–19.6)	2.71 (1.24–5.90)	6.59 (3.58–12.1)
**100–199**	**Advanced**	123.0 (70.6–214.4)	70.6 (46.7–106.8)	14.5 (8.67–24.1)	5.34 (3.46–8.23)	13.6 (11.5–16.1)
	**Less advanced**	41.5 (18.7–91.9)	23.8 (11.8–48.1)	4.87 (2.29–10.4)	1.80 (0.98–3.31)	4.57 (2.67–7.84)
**≥200**	**Advanced**	89.5 (62.1–129.0)	34.3 (18.4–63.8)	16.1 (11.2–23.1)	3.39 (1.79–6.40)	10.2 (7.63–13.7)
	**Less advanced**	30.2 (15.7–58.0)	11.5 (4.98–26.8)	5.43 (3.13–9.43)	1.14 (0.47–2.77)	3.44 (1.91–6.17)
**Overall**	**Overall**	130.0 (110.9–152.4)	49.6 (42.2–58.3)	13.4 (10.4–17.3)	4.05 (3.25–5.04)	18.7 (17.7–19.8)

### Sensitivity Analyses

When restricting the analysis to the three treatment programmes with rates of loss to follow-up below 10% (Khayelitsha, Gugulethu, Connaught), estimates of excess mortality and SMRs were somewhat lower, but the variation with time period, baseline CD4, and clinical stage was similar to that observed using all data (Tables S4 and S5). For example, in the second year on ART excess mortality in the patients group with the best prognosis was 0.15 (0.015–1.50) per 100 person-years and the SMR was 0.76 (0.18–3.10).

## Discussion

In this collaborative study of five treatment programmes in four countries in sub-Saharan Africa, the mortality of HIV-infected patients starting ART could be compared with that estimated for the corresponding non-HIV–infected general populations. In these countries, a large proportion of deaths among young and middle-aged adults are HIV-related. We found that mortality during the first 2 y of ART was more than 18 times higher than in the general population not infected by HIV. However, there was large variability between prognostic groups and over time: in patients with very low CD4 counts and advanced clinical disease, mortality was increased over 300 times in the first 3 mo of treatment, whereas in the second year of ART, patients who started with high CD4 counts and less advanced disease had mortality rates that were comparable to those estimated for non-HIV–infected individuals.

We used excess mortality rates as well as SMRs and thus took the background mortality in the general population into account. The calculation of expected numbers of deaths was restricted to people not infected with HIV, which is crucial when the prevalence of the exposure (HIV infection) in the general population is high and SMRs are large [25]. The mortality of over 13,000 patients was analyzed, including women and men, teenagers and middle-aged people, and patients with severe and less pronounced immunodeficiency. Our results should therefore be applicable to many other patients receiving ART in sub-Saharan Africa. We used estimates of non-HIV–related mortality from the WHO Global Burden of Disease project [17]. Beginning with the year 1999, WHO has been producing annual life tables for all member states. A key use of these tables is the calculation of healthy life expectancy, the basic indicator of population health published each year in the World Health Report [18].

One limitation of our study is that the reference rates for HIV-unrelated mortality are unlikely to be completely accurate for the source populations from which the HIV-infected patients originate, and that errors in the calculation of expected number of deaths are not reflected in the confidence limits of SMRs and excess mortality rates [26]. The five ART programmes included in this study are public sector scale-up programmes, which serve disadvantaged urban populations. Data from the 1970s and early 1980s suggest that adult mortality is lower in urban Africa than in rural Africa [27]. The generally lower mortality rates observed in urban settings may, however, conceal pockets of poverty and high mortality among urban dwellers [27]. Nevertheless, the use of national rates may have lead to estimates of the expected number of HIV-unrelated deaths that are too high, and SMRs and excess mortality rates that are too low. Given that reliable local mortality data are not available, we believe that the data from the Global Burden of Disease project are the best reference data available. Of note, the estimates used in this study for South Africa are in line with those from other analyses. For example, a recent modelling study of the demographic impact of HIV/AIDS in South Africa by the University of Cape Town and the South African Medical Research Council estimated that in 2006, 71% of deaths in the 15–49 y age group were due to HIV infection [28]. Similarly, a study of AIDS-related mortality in rural KwaZulu-Natal estimated that 127 of 186 deaths (68%) were attributable to AIDS in 2004 [29]. A demographic surveillance study using verbal autopsy in the Agincourt subdistrict, rural South Africa, also found that HIV and tuberculosis were the leading causes of death in people aged 15–49 y [30].

Our study has other limitations. Complete ascertainment of risk factors and deaths and complete follow-up of patients is difficult to achieve in treatment programmes in low-income countries [31],[32]. Loss to follow-up was particularly high in one programme in Malawi, however, this is probably due to a higher rate of transfer out of patients in this programme. At present we cannot distinguish between loss to follow-up and transfer to another programme; this will be remedied in the next update of the database. We used multiple imputation to deal with missing baseline CD4 cell counts and loss to follow-up. This method assumes that missing values can accurately be predicted using the available data. In other words, the probability of missing no longer depends on the missing value after taking the available data into account (“missing at random” in Rubin's terminology [33]). The plausibility of this assumption is unverifiable, but it is clear that mortality is increased in patients lost to follow-up [34]–[36], and unlikely that this can fully be captured by the clinical stage and CD4 cell count at baseline. Of note, sensitivity analyses excluding the sites with high rates of loss to follow-up from Malawi and Côte d'Ivoire gave similar results.

Follow-up was limited to 2 y in the present analyses, reflecting the relatively recent scale up of ART in sub-Saharan Africa, and it is possible that mortality will increase again in HIV-infected patients with longer duration of treatment. The short follow-up also meant that life expectancy of patients starting ART could not be examined. The ART Cohort Collaboration of HIV cohorts in Europe and North America recently estimated that life expectancy at age 35 y among patients on ART not infected through injecting drug use was 33 y [37]. These questions will be addressed in future analyses of the IeDEA databases. Finally, our analysis did not consider differences between the HIV-infected and non-HIV–infected populations other than gender and age. In industrialised countries, there are important differences in the prevalence of risk factors, for example smoking, between infected and noninfected populations. In sub-Saharan Africa, where the epidemic is generalised and transmission by heterosexual contacts, differences in lifestyle factors are unlikely to be a major source of bias.

How do these SMRs compare with other population groups at increased risk of death due to unhealthy lifestyles, occupational exposures, or chronic conditions other than HIV infection? Few data are available for sub-Saharan Africa. White South African gold miners, compared to the white male population, had an SMR of 1.3, because of excess mortality due to lung cancer, chronic obstructive lung disease, and liver cirrhosis [38]. Among male British doctors born in the 1920s, the probability of dying from any cause in middle age was three times higher in smokers than lifelong nonsmokers [39]. Similarly, an analysis of the National Alcohol Survey in the US showed that regular, heavy drinkers had mortality rates from all causes that were 2.2 times higher than those observed in lifetime abstainers [40]. The mortality of people with a body mass index (BMI) over 35 kg/m^2^ is increased by factor 1.5 to 2.5, compared to those with a BMI between 20 and 25 kg/m^2^, and a similar increase in all-cause mortality is found in physically inactive people compared to physically active individuals [41]. In a population-based study in Turin, Northern Italy, women with type 1 diabetes had an SMR for all causes of 3.4 and men an SMR of 2.0 [42]. The SMRs found in these patients and populations exposed to risk factors are thus quite comparable to those found in some of the patient groups included in our analysis.

Excess mortality was greater among men than among women. A recent analysis from the ART in Lower Income Countries (ART-LINC) collaboration found that although women are more vulnerable than men to becoming infected with HIV, they were equally or more likely than men to start ART [43]. Women were younger and started treatment at a less advanced clinical stage, which could partly explain their lower excess mortality. Gender inequities in health may affect men as well as women: traditional masculine roles cast men as taking risks, being unconcerned about their health, and not needing help or healthcare [44]. Conventional views of gender inequality might have made it easier for women than men in some settings to become engaged with HIV diagnosis and treatment services [43],[45],[46]. Clearly, continued efforts are needed to empower women and secure their rights to treatment and care for HIV infection. However, more attention needs to be paid to HIV-infected men.

Although some HIV-infected patients starting ART in sub-Saharan Africa experienced mortality rates that were comparable with those experienced by other patients with a chronic condition, early mortality in adults starting ART continues to be high in sub-Saharan Africa [47]. Many patients start treatment late, with a history of AIDS defining illnesses and low CD4 cell counts. Leading causes of death include tuberculosis, acute sepsis, cryptococcal meningitis, malignancies, and wasting syndrome [47]. Of note, the Starting Antiretrovirals at three Points in Tuberculosis (SAPIT) trial recently showed that mortality among patients co-infected with tuberculosis and HIV can be reduced by 55% if ART is provided with TB treatment [48]. Although our study cannot determine the CD4 cell count when ART should be started in order to minimise mortality, much of the excess mortality observed in our study would probably be preventable with timely initiation of ART. Further expansion of public health strategies to increase access to ART in sub-Saharan Africa is therefore urgently needed. In collaboration with the Global Burden of Disease project, the IeDEA network will continue to monitor mortality of HIV-infected patients starting ART and compare their mortality to that of the general population not infected by HIV.

## Supporting Information

Table S1 Age- and sex-specific HIV-unrelated mortality per 100 population in Côte d'Ivoire, Malawi, Zimbabwe, and South Africa, 2004. Data from the Global Burden of Disease study [17],[20].
(0.05 MB DOC)

Table S2 Excess mortality per 100 person-years for months 1–24 by baseline CD4 count and clinical stage of disease, and by sex and age group.
(0.06 MB DOC)

Table S3 SMRs for months 1–24 by baseline CD4 count and clinical stage of disease, and by sex and age group.
(0.06 MB DOC)

Table S4 Excess mortality per 100 person-years by time period on ART, baseline CD4 count, and clinical stage of disease in the three ART programmes with low rates of loss to follow-up (Connaught, Gugulethu, Khayelitsha).
(0.04 MB DOC)

Table S5 SMRs by time period on ART, baseline CD4 count, and clinical stage of disease in the three ART programmes with low rates of loss to follow-up (Connaught, Gugulethu, Khayelitsha).
(0.04 MB DOC)

## Abbreviations

ART: antiretroviral therapy
eHR: excess hazard ratio
IQR: interquartile range
SMR: standardised mortality ratio
WHO: World Health Organization
