# Supplementary material for: Mortality of HIV-Infected Patients Starting Antiretroviral Therapy in Sub-Saharan Africa: Comparison with HIV-Unrelated Mortality
Source: PLoS Med. 2009 Apr 28;6(4):e1000066. doi: 10.1371/journal.pmed.1000066 (PMC2667633; doi:10.1371/journal.pmed.1000066)
Supplement: Table S1 — Age- and sex-specific HIV-unrelated mortality per 100 population in Côte d'Ivoire, Malawi, Zimbabwe, and South Africa, 2004. Data from the Global Burden of Disease study [17],[20]. (0.05 MB DOC) [file pmed.1000066.s001.doc]

**Table S1 – Age- and sex-specific HIV-unrelated mortality per 100 population in Côte d’Ivoire, Malawi, Zimbabwe and South Africa, 2004**Data from the Global Burden of Disease study.

|  | Côte d’Ivoire | |  | Malawi |  |  | Zimbabwe | |  | South Africa | |
| --- | --- | --- | --- | --- | --- | --- | --- | --- | --- | --- | --- |
| Age group | Women | Men |  | Women | Men |  | Women | Men |  | Women | Men |
| 15-29 | 0.255 | 0.182 |  | 0.249 | 0.165 |  | 0.198 | 0.226 |  | 0.184 | 0.244 |
| 20-24 | 0.372 | 0.513 |  | 0.364 | 0.322 |  | 0.260 | 0.411 |  | 0.241 | 0.383 |
| 25-29 | 0.427 | 0.710 |  | 0.416 | 0.411 |  | 0.298 | 0.487 |  | 0.273 | 0.435 |
| 30-39 | 0.480 | 0.892 |  | 0.466 | 0.512 |  | 0.379 | 0.599 |  | 0.333 | 0.537 |
| 35-39 | 0.569 | 1.119 |  | 0.553 | 0.667 |  | 0.524 | 0.748 |  | 0.438 | 0.713 |
| 40-44 | 0.678 | 1.359 |  | 0.660 | 0.884 |  | 0.734 | 0.929 |  | 0.588 | 0.978 |
| 45-49 | 0.856 | 1.669 |  | 0.837 | 1.198 |  | 1.003 | 1.221 |  | 0.796 | 1.337 |
| 50-54 | 1.199 | 2.118 |  | 1.177 | 1.660 |  | 1.378 | 1.686 |  | 1.110 | 1.831 |
| 55-59 | 1.797 | 2.958 |  | 1.768 | 2.449 |  | 1.874 | 2.463 |  | 1.583 | 2.560 |
| 60-64 | 2.469 | 3.906 |  | 2.439 | 3.406 |  | 2.570 | 3.414 |  | 2.240 | 3.569 |
| 65-69 | 3.946 | 5.463 |  | 3.911 | 4.942 |  | 3.659 | 4.879 |  | 3.427 | 5.019 |
| 70-74 | 6.268 | 8.126 |  | 6.229 | 7.480 |  | 5.510 | 7.294 |  | 5.428 | 7.395 |
