# Supplementary material for: Mortality of HIV-Infected Patients Starting Antiretroviral Therapy in Sub-Saharan Africa: Comparison with HIV-Unrelated Mortality
Source: PLoS Med. 2009 Apr 28;6(4):e1000066. doi: 10.1371/journal.pmed.1000066 (PMC2667633; doi:10.1371/journal.pmed.1000066)
Supplement: Table S2 — Excess mortality per 100 person-years for months 1–24 by baseline CD4 count and clinical stage of disease, and by sex and age group. (0.06 MB DOC) [file pmed.1000066.s002.doc]

**Table S2 – Excess mortality per 100 person-years for months 1-24 by baseline CD4 count and clinical stage of disease, and by sex and age group**

|  |  | Age (years) at start of HAART | | | | |
| --- | --- | --- | --- | --- | --- | --- |
| CD4 count (cells/μL) | Clinical stage | 16-29 | 30-39 | 40-49 | ≥ 50 | Overall |
| Women |  |  |  |  |  |  |
| < 25 | Advanced | 15.1 (11.5-19.8) | 16.3 (14.2-18.7) | 16.5 (14.0-19.5) | 19.4 (15.3-24.5) | 16.0 (13.7-18.6) |
|  | Less advanced | 4.3 (2.2-8.3) | 4.6 (2.6- 8.3) | 4.7 (2.5-8.8) | 5.5 (3.1-9.8) | 4.5 (2.5-8.3) |
| 25-49 | Advanced | 10.4 (7.1-15.2) | 11.2 (9.3-13.6) | 11.3 (9.0-14.3) | 13.3 (9.9-17.9) | 11.0 (8.6-14.1) |
|  | Less advanced | 2.9 (1.4-6.1) | 3.2 (1.7-5.8) | 3.2 (1.7-6.2) | 3.8 (2.0-7.0) | 3.1 (1.7-5.9) |
| 50-99 | Advanced | 6.4 (3.9-10.5) | 6.9 (5.4-8.9) | 7.0 (5.1-9.6) | 8.2 (5.5-12.1) | 6.8 (4.8-9.6) |
|  | Less advanced | 1.8 (0.79-4.2) | 2.0 (1.0-3.8) | 2.0 (0.95-4.1) | 2.3 (1.1-4.7) | 1.9 (0.94-4.0) |
| 100-199 | Advanced | 4.2 (2.8-6.4) | 4.6 (3.8-5.5) | 4.6 (3.7-5.8) | 5.4 (3.9-7.5) | 4.5 (3.5-5.9) |
|  | Less advanced | 1.2 (0.57-2.5) | 1.3 (0.71-2.4) | 1.3 (0.68-2.5) | 1.5 (0.82-2.9) | 1.3 (0.67-2.4) |
| ≥ 200 | Advanced | 3.1 (2.6-3.8) | 3.4 (2.4-4.7) | 3.4 (2.5-4.6) | 4.0 (2.9-5.6) | 3.3 (2.6-4.3) |
|  | Less advanced | 0.89 (0.49-1.6) | 0.96 (0.52-1.8) | 0.97 (0.51-1.8) | 1.1 (0.33-2.1) | 0.95 (0.53-1.7) |
| Overall | Overall | 6.1 (4.9-7.4) | 6.3 (5.4-7.2) | 6.0 (4.9-7.4) | 6.5 (5.1-8.4) | 6.2 (5.4-7.1) |
|  |  |  |  |  |  |  |
| Men |  |  |  |  |  |  |
| < 25 | Advanced | 18.4 (12.2-27.8) | 19.9 (16.9-23.5) | 20.2 (16.0-25.4) | 23.6 (17.5-31.9) | 20.0 (16.0-25.1) |
|  | Less advanced | 5.2 (2.5-11.0) | 5.6 (3.1-10.2) | 5.7 (3.0-11.0) | 6.7 (3.6-12.4) | 5.7 (3.1-10.6) |
| 25-49 | Advanced | 12.6 (7.6-21.2) | 13.7 (10.5-17.8) | 13.8 (10.0-19.0) | 16.2 (11.1-23.7) | 13.8 (10.0-19.0) |
|  | Less advanced | 3.6 (1.6-8.0) | 3.9 (2.0-7.3) | 3.9 (1.9-7.9) | 4.6 (2.4-9.0) | 3.9 (2.0-7.7) |
| 50-99 | Advanced | 7.8 (4.1-14.9) | 8.4 (5.8-12.3) | 8.5 (5.5-13.3) | 10.0 (6.0-16.6) | 8.5 (5.5-13.3) |
|  | Less advanced | 2.2 (0.87-5.6) | 2.4 (1.1-5.0) | 2.4 (1.1-5.4) | 2.8 (1.3-6.2) | 2.4 (1.1-5.2) |
| 100-199 | Advanced | 5.2 (3.0-9.0) | 5.6 (4.2-7.5) | 5.6 (4.0-8.0) | 6.6 (4.3-10.2) | 5.6 (3.9-8.1) |
|  | Less advanced | 1.5 (0.63-3.4) | 1.6 (0.83-3.0) | 1.6 (0.79-3.2) | 1.9 (0.94-3.7) | 1.6 (0.81-3.2) |
| ≥ 200 | Advanced | 3.8 (2.9-5.0) | 4.1 (3.2-5.3) | 4.2 (3.3-5.3) | 4.9 (3.6-6.6) | 4.2 (3.4-5.2) |
|  | Less advanced | 1.1 (0.57-2.0) | 1.2 (0.66-2.1) | 1.2 (0.64-2.2) | 1.4 (0.78-2.5) | 1.2 (0.67-2.1) |
| Overall | Overall | 8.5 (6.1-11.7) | 8.8 (7.8-9.9) | 8.5 (6.9-10.4) | 9.2 (7.0-12.0) | 8.7 (7.3-10.3) |
