# Supplementary material for: Mortality of HIV-Infected Patients Starting Antiretroviral Therapy in Sub-Saharan Africa: Comparison with HIV-Unrelated Mortality
Source: PLoS Med. 2009 Apr 28;6(4):e1000066. doi: 10.1371/journal.pmed.1000066 (PMC2667633; doi:10.1371/journal.pmed.1000066)
Supplement: Table S3 — SMRs for months 1–24 by baseline CD4 count and clinical stage of disease, and by sex and age group. (0.06 MB DOC) [file pmed.1000066.s003.doc]

**Table S3 – Standardized mortality ratios (SMRs) for months 1-24 by baseline CD4 count and clinical stage of disease, and by sex and age group**

|  |  | Age (years) at start of HAART | | | | |
| --- | --- | --- | --- | --- | --- | --- |
| CD4 count (cells/μL) | Clinical stage | 16-29 | 30-39 | 40-49 | ≥ 50 | Overall |
| Women |  |  |  |  |  |  |
| < 25 | Advanced | 79.5 (64.4-98.2) | 60.5 (48.0-76.4) | 38.6 (31.1-47.8) | 24.1 (19.2-30.2) | 56.6 (46.7-68.6) |
|  | Less advanced | 26.5 (15.1-46.6) | 20.2 (11.4-35.6) | 12.9 (7.22-22.9) | 8.02 (4.98-12.9) | 18.6 (10.6-32.8) |
| 25-49 | Advanced | 55.9 (43.0-72.5) | 42.5 (35.6-50.8) | 27.1 (22.0-33.4) | 16.9 (13.3-21.5) | 37.6 (31.3-45.0) |
|  | Less advanced | 18.6 (10.6-32.8) | 14.2 (8.34-24.1) | 9.03 (5.17-15.8) | 5.63 (3.55-8.94) | 12.4 (7.05-21.7) |
| 50-99 | Advanced | 34.9 (24.5-49.7) | 26.6 (21.8-32.4) | 16.9 (13.1-22.0) | 10.6 (7.90-14.1) | 23.3 (18.9-28.7) |
|  | Less advanced | 11.6 (6.2-21.9) | 8.86 (5.07-15.5) | 5.64 (3.10-10.3) | 3.52 (2.11-5.88) | 7.65 (4.24-13.8) |
| 100-199 | Advanced | 24.3 (19.1-31.1) | 18.5 (16.1-21.4) | 11.8 (10.0-14.0) | 7.37 (5.79-9.37) | 15.9 (13.8-18.4) |
|  | Less advanced | 8.12 (4.74-13.9) | 6.18 (3.76-10.1) | 3.94 (2.34-6.63) | 2.46 (1.59-3.80) | 5.24 (3.09-8.89) |
| ≥ 200 | Advanced | 18.5 (14.1-24.3) | 14.1 (9.62-20.7) | 8.98 (6.56-12.3) | 5.60 (3.99-7.87) | 11.9 (8.46-16.6) |
|  | Less advanced | 6.17 (3.45-11.0) | 4.70 (2.48-8.90) | 2.99 (1.62-5.54) | 1.87 (1.10-3.18) | 3.91 (2.12-7.20) |
| Overall | Overall | 33.2 (28.2-39.1) | 24.4 (19.3-30.8) | 14.8 (11.6-18.9) | 8.49 (6.65-10.8) | 21.2 (16.7-26.9) |
|  |  |  |  |  |  |  |
| Men |  |  |  |  |  |  |
| < 25 | Advanced | 65.5 (42.2-101.8) | 49.9 (36.9-67.4) | 31.8 (22.5-44.9) | 19.8 (15.3-25.7) | 38.9 (29.8-50.8) |
|  | Less advanced | 21.8 (10.6-45.1) | 16.6 (8.71-31.8) | 10.6 (5.36-21.0) | 6.61 (3.82-11.4) | 12.8 (6.78-24.2) |
| 25-49 | Advanced | 46.0 (27.7-76.4) | 35.0 (25.3-48.6) | 22.3 (15.0-33.2) | 13.9 (9.95-19.5) | 25.8 (19.2-34.6) |
|  | Less advanced | 15.3 (7.21-32.6) | 11.7 (6.13-22.3) | 7.44 (3.72-14.9) | 4.64 (2.62-8.23) | 8.49 (4.45-16.2) |
| 50-99 | Advanced | 28.8 (15.7-52.8) | 21.9 (14.5-33.1) | 14.0 (8.59-22.7) | 8.70 (5.59-13.5) | 16.0 (10.9-23.5) |
|  | Less advanced | 9.59 (4.14-22.2) | 7.30 (3.59-14.9) | 4.65 (2.17-9.99) | 2.90 (1.50-5.60) | 5.26 (2.58-10.7) |
| 100-199 | Advanced | 20.1 (12.2-32.9) | 15.3 (11.3-20.7) | 9.74 (6.72-14.1) | 6.07 (4.35-8.47) | 11.0 (8.26-14.5) |
|  | Less advanced | 6.69 (3.22-13.9) | 5.09 (2.75-9.42) | 3.25 (1.67-6.30) | 2.02 (1.17-3.50) | 3.60 (1.93-6.72) |
| ≥ 200 | Advanced | 15.3 (10.3-22.7) | 11.6 (8.26-16.3) | 7.40 (5.35-10.3) | 4.62 (3.57-5.96) | 8.15 (6.09-10.9) |
|  | Less advanced | 5.09 (2.54-10.2) | 3.87 (2.00-7.50) | 2.47 (1.27-4.80) | 1.54 (0.90-2.64) | 2.68 (1.43-5.03) |
| Overall | Overall | 31.2 (22.0-44.2) | 22.9 (17.7-29.6) | 13.9 (10.0-19.2) | 7.97 (6.25-10.2) | 16.0 (12.6-20.3) |
