# Supplementary material for: Mortality of HIV-Infected Patients Starting Antiretroviral Therapy in Sub-Saharan Africa: Comparison with HIV-Unrelated Mortality
Source: PLoS Med. 2009 Apr 28;6(4):e1000066. doi: 10.1371/journal.pmed.1000066 (PMC2667633; doi:10.1371/journal.pmed.1000066)
Supplement: Table S4 — Excess mortality per 100 person-years by time period on ART, baseline CD4 count, and clinical stage of disease in the three ART programmes with low rates of loss to follow-up (Connaught, Gugulethu, Khayelitsha). (0.04 MB DOC) [file pmed.1000066.s004.doc]

**Table S4 – Excess mortality per 100 person-years by time period on ART, baseline CD4 count and clinical stage of disease in the three ART programmes with low rates of loss to follow-up (Connaught, Gugulethu, Khayelitsha)**

|  |  |  | **Time period (months)** | | | | |
| --- | --- | --- | --- | --- | --- | --- | --- |
| **CD4 count (cells/μL)** | **Clinical stage** |  | **1-3** | **4-6** | **7-12** | **13-24** | **Overall (1-24)** |
| < 25 | Advanced |  | 52.98 (38.45-73.02) | 10.35 (6.27-17.09) | 6.88 (4.54-10.42) | 4.21 (2.96-6.00) | 15.7 (14.4-17.2) |
|  | Less advanced |  | 12.89 (5.63-29.53) | 2.52 (0.91-6.96) | 1.67 (0.64-4.37) | 1.03 (0.56-1.87) | 3.79 (1.71-8.40) |
| 25-49 | Advanced |  | 29.16 (22.58-37.67) | 11.41 (6.46-20.16) | 4.91 (3.25-7.43) | 1.60 (0.75-3.44) | 9.84 (8.77-11.05) |
|  | Less advanced |  | 7.10 (3.29-15.31) | 2.78 (1.12-6.91) | 1.20 (0.44-3.26) | 0.39 (0.15-0.98) | 2.37 (1.08-5.20) |
| 50-99 | Advanced |  | 13.84 (7.54-25.41) | 6.17 (2.72-14.01) | 3.35 (1.67-6.73) | 2.38 (0.90-6.30) | 5.69 (3.78-8.56) |
|  | Less advanced |  | 3.37 (1.27-8.93) | 1.50 (0.47-4.81) | 0.82 (0.24-2.76) | 0.58 (0.13-2.55) | 1.37 (0.52-3.60) |
| 100-199 | Advanced |  | 9.03 (4.20-19.45) | 5.28 (4.20-6.64) | 2.09 (1.33-3.30) | 1.35 (0.53-3.46) | 3.81 (2.97-4.90) |
|  | Less advanced |  | 2.20 (0.68-7.15) | 1.28 (0.54-3.03) | 0.51 (0.24-1.10) | 0.33 (0.089-1.21) | 0.92 (0.39-2.17) |
| ≥ 200 | Advanced |  | 12.03 (9.19-15.75) | 5.30 (3.50-8.04) | 3.45 (1.80-6.61) | 0.62 (0.10-3.64) | 4.79 (3.38-6.79) |
|  | Less advanced |  | 2.93 (1.24-6.90) | 1.29 (0.79-2.10) | 0.84 (0.25-2.87) | 0.15 (0.015-1.50) | 1.15 (0.43-3.07) |
| **Overall** | **Overall** |  | 19.67 (17.26-22.43) | 6.81 (6.02-7.70) | 3.47 (2.65-4.53) | 2.03 (1.50-2.73) | 6.35 (5.44-7.40) |
