# Supplementary material for: Mortality of HIV-Infected Patients Starting Antiretroviral Therapy in Sub-Saharan Africa: Comparison with HIV-Unrelated Mortality
Source: PLoS Med. 2009 Apr 28;6(4):e1000066. doi: 10.1371/journal.pmed.1000066 (PMC2667633; doi:10.1371/journal.pmed.1000066)
Supplement: Table S5 — SMRs by time period on ART, baseline CD4 count, and clinical stage of disease in the three ART programmes with low rates of loss to follow-up (Connaught, Gugulethu, Khayelitsha). (0.04 MB DOC) [file pmed.1000066.s005.doc]

**Table S5 - Standardized mortality ratios (SMRs) by time period on ART, baseline CD4 count and clinical stage of disease in the three ART programmes with low rates of loss to follow-up (Connaught, Gugulethu, Khayelitsha)**

|  |  |  | **Time period (months)** | | | | |
| --- | --- | --- | --- | --- | --- | --- | --- |
| **CD4 count (cells/μL)** | **Clinical stage** |  | **1-3** | **4-6** | **7-12** | **13-24** | **Overall (1-24)** |
| < 25 | Advanced |  | 499.0 (369.3-674.2) | 101.7 (63.6-162.5) | 34.2 (23.5-49.7) | 10.6 (7.52-14.9) | 48.2 (40.9-56.9) |
|  | Less advanced |  | 152.9 (86.3-271.0) | 31.2 (14.7-65.9) | 10.5 (5.24-21.0) | 3.24 (2.12-4.97) | 15.3 (6.75-34.8) |
| 25-49 | Advanced |  | 271.6 (213.1-346.2) | 111.0 (63.7-193.3) | 25.0 (17.4-36.1) | 4.70 (2.58-8.58) | 29.2 (24.2-35.3) |
|  | Less advanced |  | 83.2 (49.8-138.9) | 34.0 (17.2-67.4) | 7.67 (3.59-16.4) | 1.44 (0.73-2.83) | 9.29 (4.06-21.3) |
| 50-99 | Advanced |  | 131.4 (74.2-232.9) | 61.6 (28.9-131.3) | 17.5 (9.47-32.5) | 6.53 (2.87-14.8) | 17.6 (12.2-25.5) |
|  | Less advanced |  | 40.3 (19.5-83.0) | 18.9 (7.66-46.5) | 5.37 (2.14-13.5) | 2.00 (0.65-6.11) | 5.61 (2.23-14.1) |
| 100-199 | Advanced |  | 88.9 (44.9-176.3) | 55.2 (45.5-67.0) | 11.9 (7.44-18.9) | 4.56 (2.43-8.56) | 13.0 (10.6-15.9) |
|  | Less advanced |  | 27.3 (11.2-66.1) | 16.9 (9.60-29.8) | 3.63 (2.06-6.39) | 1.40 (0.66-2.98) | 4.13 (1.80-9.44) |
| ≥ 200 | Advanced |  | 116.2 (89.4-151.0) | 55.0 (36.1-83.8) | 19.8 (11.4-34.4) | 2.47 (0.84-7.30) | 17.1 (10.1-29.1) |
|  | Less advanced |  | 35.6 (18.9-67.1) | 16.8 (12.0-23.7) | 6.06 (2.40-15.3) | 0.76 (0.18-3.10) | 5.45 (1.75-16.9) |
| **Overall** | **Overall** |  | 141.5 (107.2-186.9) | 52.5 (41.3-66.9) | 14.2 (9.87-20.5) | 4.50 (3.14-6.45) | 20.1 (18.5-21.8) |
